# Safety considerations in the treatment with anti-CGRP(R) monoclonal antibodies in patients with migraine

**DOI:** 10.3389/fneur.2024.1387044

**Published:** 2024-04-29

**Authors:** Britt W. H. Van Der Arend, Nancy Van Veelen, Joëlle E. T. De Ruijter, Michael H. Olsen, Antoinette MaassenVanDenBrink, Gisela M. Terwindt

**Affiliations:** ^1^Department of Neurology, Leiden University Medical Center, Leiden, Netherlands; ^2^Department of Internal Medicine, Erasmus MC University Medical Center, Rotterdam, Netherlands; ^3^Department of Internal Medicine, Holbaek Hospital, Holbæk, Denmark; ^4^Department of Regional Health Research, University of Southern Denmark, Odense, Denmark

**Keywords:** migraine, CGRP, monoclonals, cardiovascular risk, adverse events, headache, safety, treatment

## Abstract

**Background:**

Anti-CGRP-(receptor-)monoclonal antibodies (anti-CGRP(R)-mAbs) represent a novel class of drugs for migraine treatment, but their long-term cerebrovascular and cardiovascular (CV) safety warrants further examination.

**Methods:**

In this observational cohort study we assessed the CV safety for erenumab and fremanezumab in a real-world setting during a follow-up period of at least 1 year. Patients with hypertension or CV history were excluded. We conducted ECGs and collected clinical data at treatment initiation and thereafter every 3 months, including liver and kidney function, lipid-, electrolyte-and glucose levels.

**Results:**

Among patients receiving erenumab (*n* = 101) or fremanezumab (*n* = 92), 3.1% (6/193) developed abnormal ECGs or CV adverse events. Of these, three (1.6%) experienced moderate to severe CV adverse events (cerebellar stroke, spontaneous coronary artery dissection, and pericarditis) and discontinued treatment. The remaining three (1.6%) developed non-threatening ECG abnormalities without physical complaints. No significant changes were observed in liver and kidney function, lipid-, electrolyte-, or glucose levels.

**Discussion:**

We observed CV events in 1.6% of patients with 1.5-year follow-up of anti-CGRP(R)-mAbs treatment. We advise awareness regarding CV events in patients with migraine undergoing CGRP-targeted treatment, not as a confirmation of increased risk but as a proactive measure to address potential multifactorial influences.

## Introduction

Over the past few years, monoclonal anti-Calcitonin Gene-Related Peptide-(Receptor-)monoclonal Antibodies (anti-CGRP(R)-mAbs) have emerged as a promising prophylactic treatment for migraine (1–3). These antibodies bind to either the CGRP ligand or its receptor, thereby blocking its actions. While the efficacy of anti-CGRP(R)-mAbs in preventing migraines has been established, it is crucial to consider the potential physiological implications associated with their prolonged usage. This necessity arises from the multifaceted role of CGRP in various biological processes within the human body (4).

CGRP, a potent systemic vasodilator capable of reducing blood pressure (BP), and exerting both chronotropic and inotropic effects on the heart, plays a critical role in maintaining the cerebro-and cardiovascular (CV) homeostasis (5). In theory, anti-CGRP(R)-mAbs may diminish the protective effect of CGRP on CV-infarct size and increase the risk of heart failure (5–7). Notably, migraine itself is an independent CV risk factor, further emphasizing the need for a comprehensive understanding of the CV implications of anti-CGRP(R)-mAbs (8–10). Moreover, as CGRP can be found in the digestive tract, lungs, kidney, liver and adipose tissue as well, other regulatory systems may be affected by the neuropeptide, although less is known about the exact sites of action and underlying mechanisms (4, 11).

*In vitro* studies demonstrated that erenumab inhibited CGRP-mediated vasodilation, while not interacting with other vasoactive compounds (12). Furthermore, work in mice demonstrated that treatment with a CGRP receptor antagonist worsened cerebral ischemic outcomes (6), and rat studies demonstrated that cardiac ischemic outcome is worsened by the blocking of the CGRP receptor (7). Thus, these animal studies indicate an important role of CGRP in preserving tissue during ischemic conditions. A handful of studies have evaluated the effect of erenumab *in vivo* and yielded mixed results, with one study that did not find any alterations in vasomotor reactivity or flow-mediated dilation in patients with migraine, while another demonstrated that it did affect trigeminovascular reactivity by a decrease in capsaicin-induced dermal blood flow (13, 14).

Recent studies, including a meta-analysis of 19 randomized controlled trials and the analysis of the US FDA Adverse Event Reporting System (FAERS) database, aimed to evaluate the safety and tolerability of monoclonal antibodies and gepants targeting the CGRP pathway (15, 16). While these RCTs did not reveal differences in serious adverse events between active treatments and placebo, they were limited by their short-term nature of 3 to 6 months, their focus on solely SAEs, or lack of investigation into laboratory or ECG findings (16). Furthermore, the FAERS database, while reporting also low frequency SAEs, has limitations due to a short 6-month follow-up period and reliance on only self-reported adverse events, which likely leads to underreporting (15).

Earlier, a case report highlighted a CV-event that occurred 5 months after starting erenumab (17). Furthermore, during an open-label extension study a CV related death was reported (18). These CV-related AEs are particularly noteworthy since clinical trials typically focus on enrolling patients with migraine who need to be generally in good health. Therefore, other reviews have emphasized the importance of incorporating real-world data and post-marketing surveillance studies to validate and expand upon these trial results (19).

Of particular concern are post-marketing case reports of elevated BP associated with erenumab, raising questions about CV safety (20). An independent study found an average increase of 5.2 mm Hg in systolic BP and 3.5 mm Hg in diastolic BP after starting anti-CGRP(R)-mAbs erenumab or fremanezumab in patients with migraine (21). Studies with clinical data collected from independent researchers and with longer follow-up time seem important to obtain a more comprehensive insight and understanding of individual responses and side-effects.

In this observational cohort study we assessed the safety of erenumab and fremanezumab regarding CV safety by assessing CV events and ECGs during a period of at least 1 year of treatment with anti-CGRP(R)-mAbs in a real world setting.

## Methods

All patients with migraine who received treatment with either erenumab or fremanezumab at the Leiden Headache Center were considered eligible for inclusion. Exclusion criteria were established based on elevated risks of cardiovascular disease (CVD), and included hypertension at baseline, a medical history of hypertension and a prior CV event. At the time of this study we defined hypertension as a systolic BP ≥140 mmHg and/or a diastolic BP ≥90 mmHg in accordance with the 2018 ESC/ESH guidelines (22). In addition, we excluded patients that were previously treated with an anti-CGRP(R)-mAb and thus had no distinct baseline period. After one baseline month patients started treatment with either erenumab or fremanezumab. Due to the restricted availability of anti-CGRP(R)-mAbs in the Netherlands at the time of inclusion, all patients had to have at least 6 monthly migraine days (MMD) and failed on ≥4 migraine prophylactic treatments, including at least candesartan, beta-blockers, valproate and topiramate. Treatment failure was defined as ineffective treatment, discontinuation because of side effects or ineligibility because of contra-indications. Migraine diagnosis was made by a neurology resident in consultation with a neurologist with headache expertise, or by neurologists themselves, based on the International Classification of Headache Disorders (ICHD)-3 criteria (23). Patients with a second headache diagnosis other than tension-type headache were excluded. Approval for this study was obtained from the LUMC Medical Ethical Committee who declared no ethical concerns.

### Treatment

All patients started treatment with either erenumab 70 mg or fremanezumab 225 mg, administered subcutaneously once every 4 weeks. Patients administered the initial injection themselves under supervision of a physician or a headache nurse, and subsequent injections were administered at home. After 3 months, patients had a consultation with their treating physician, after which the erenumab dose was optionally increased to 140 mg for the subsequent 3 months based on a joint decision between patient and physician mainly taking the (side-)effects into account. As there is a strict policy in the Netherlands regarding medication overuse headache (MOH), and polypharmacy is not part of Dutch clinical practice, none of the patients used additional prophylactic treatment simultaneously with erenumab or fremanezumab or suffered from additional MOH. Thus, other preventive medication was tapered off, including a wash-out period of 1 month before anti-CGRP(R)-mAbs were administered.

### Data collection

Patients had a consultation at the Leiden Headache Center at the start of treatment (baseline) and thereafter every 3 months (3, 6, 9, 12, 15, and 18 months) until treatment was discontinued. All CV adverse events were documented in the electronic patient file over the entire follow-up period (18 months) and subsequently discussed with both a neurologist (GMT) and a cardiologist for further evaluation and management. For all patients that developed CV adverse events, the SCORE2 prediction model was used to estimate 10-year fatal and non-fatal cardiovascular risk at baseline, using the table of moderate CV risk due to their diagnosis of migraine (24). Factors included in this prediction model are sex, age, systolic BP, smoking status and non-HDL cholesterol.

Laboratory values were collected from the electronic patient records at each timepoint, with a minimum follow-up of 12 months. The collected laboratory values included electrolytes (sodium, potassium, urea), glucose, liver function [alkaline phosphatase (ALP), alanine aminotransferase (ALAT), aspartate aminotransferase (ASAT) and gamma-glutamyl transferase (GGT)], kidney function [creatinine and the estimated glomerular filtration rate (eGFR)] and lipids [cholesterol, low-density lipoprotein (LDL), high-density lipoprotein (HDL) and triglycerides]. The eGFR was calculated using the Chronic Kidney Disease Epidemiology Collaboration (CKD-EPI) equation.

ECG and blood pressure values were collected from the electronic patient records at each timepoint, with a minimum follow-up of 12 months. An automated ECG interpretation was generated and assessed by the treating physician. Heart rhythm, heart rate, electrical axis, conduction intervals, P-wave morphology, QRS complex morphology, and ST-T segment changes were documented. If any abnormal findings were detected, consultation with a cardiologist was sought for further interpretation and evaluation. The evaluations and interpretations of the ECGs were documented in the electronic patient file and later extracted for the database (BvdA and JdR). In the case of abnormal ECG findings, they underwent a meticulous review for a second time, with the assistance of a cardiologist for in-depth analysis and assessment.

### Statistics

Our primary outcome was the occurrence of CV adverse events during treatment with anti-CGRP(R)-mAbs. Secondary outcomes were the change in ECG values (HR, PR-interval, QRS-complex and QTc-interval) and clinical blood laboratory values and over time compared to baseline. Sample size was based on the available data. Baseline characteristics included sex, age, headache diagnosis, baseline monthly headache days (MHD), monthly migraine days (MMD) and systolic and diastolic BP and were summarized using means, standard deviations, frequencies and proportions. Descriptive statistics were employed to analyze the data on CV adverse events. The descriptive analysis included determining the frequency and prevalence of adverse events.

To identify potential outliers, a principal component analysis (PCA) was conducted on all laboratory values. For all laboratory and ECG values, a linear mixed model was fitted with time, sex, age, body mass index and treatment (and in case of erenumab including the dosages 70 and 140 mg) as fixed effects and the patient as a random effect, to adjust for potential confounders. For serum creatinine and eGFR, the systolic BP and diastolic BP values measured at baseline were added to the model as additional covariates. For all linear mixed models the assumptions were checked. All missing data is assumed to be missing at random, except for patients who discontinued treatment. To address this issue, complete case analyses were additionally performed. This approach was used for laboratory and ECG values in addition to the main analyses.

We used the Bonferroni correction to counteract the multiple comparisons (*n* = 20) and assessed each hypothesis at *α* = 0.05/20 = 0.003. The corrected *p*-values are displayed. The analyses were performed in R version 4.2.1 and the *lme4* package was used to fit linear mixed-effects models.

## Results

**Figure 1:**
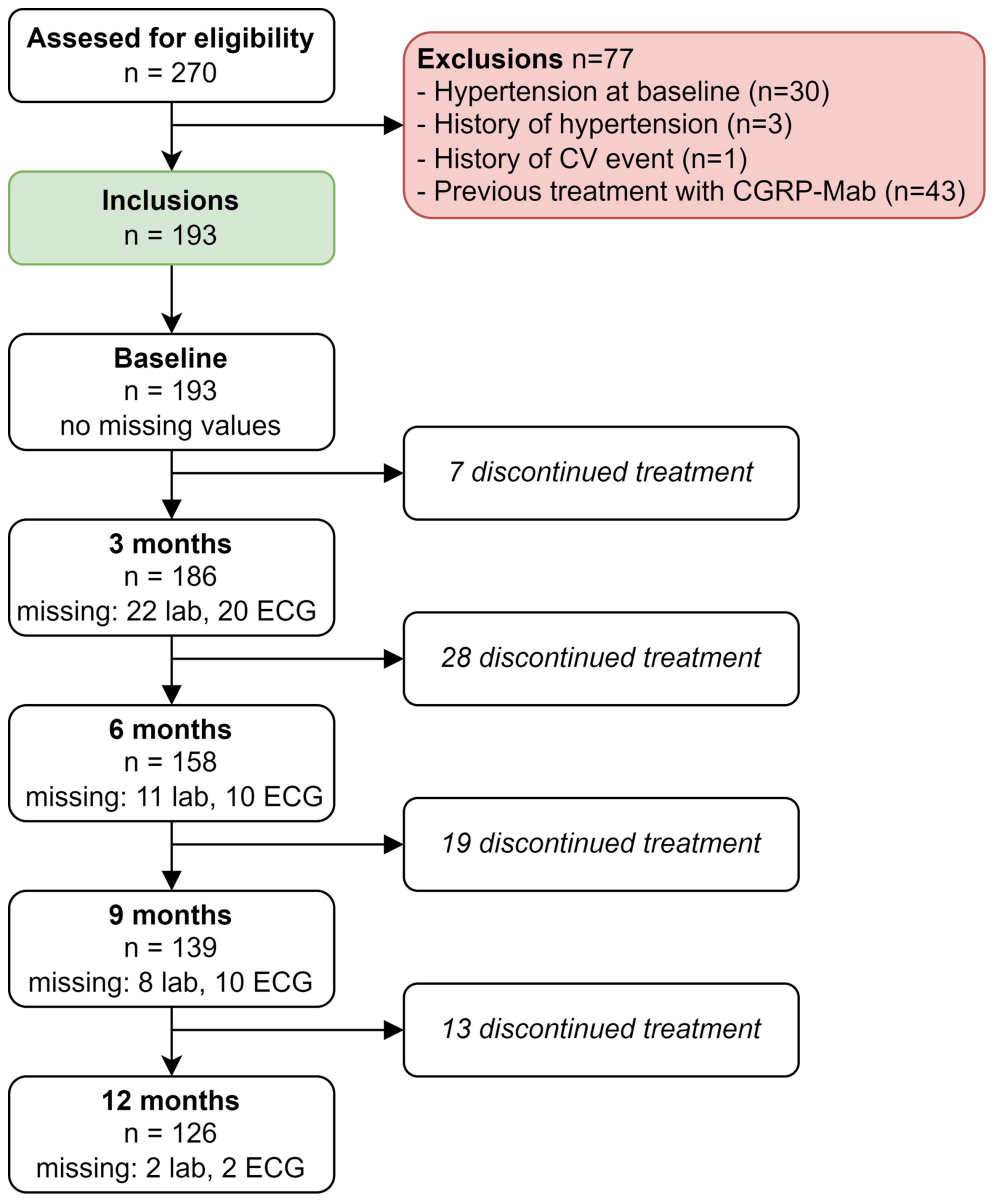
Flowchart of inclusion process.

Over a span of 3 years, from mid-November 2018 to mid-November 2021, a comprehensive assessment was carried out to establish study eligibility, involving a total of 270 patients. The study’s inclusion process is visually presented in Figure 1. Among the patients considered for participation, a total of *n* = 77 were excluded. Primary reasons for exclusion were previous treatment with anti-CGRP(R)-mAbs (*n* = 43) or an increased CV risk due to hypertension at baseline (*n* = 30), a history of hypertension (*n* = 3) or a history of CV events (*n* = 1).

A total of 193 patients with migraine started treatment with either erenumab (*n* = 101) or fremanezumab (*n* = 92). Among the patients treated with erenumab, 82/101 (81%) were female, the average age was 43 years and 43/101 (43%) patients had migraine with aura. Among the patients treated with fremanezumab, 76/92 (83%) were women, the average age was 43 years and 32/92 (35%) patients had migraine with aura. None of the patients had hypertension or were smoking at baseline. Baseline characteristics for both treatment groups are presented in Table 1.

**Table 1:** Patient characteristics for patients treated with erenumab or fremanezumab.

	Total (*n* = 193)	Erenumab (*n* = 101)	Fremanezumab (*n* = 92)
Women, n (%)	158 (81.9)	82 (80.8)	76 (82.6)
Age, mean ± SD (years)	43 ± 13.2	43 ± 12.7	43 ± 13.7
Chronic migraine, n (%)	106 (54.9)	53 (52.5)	53 (57.6)
Migraine with aura, n (%)	75 (38.9)	43 (42.6)	32 (34.8)
MMD baseline, mean ± SD	13.9 ± 5.9	14.1 ± 5.8	13.8 ± 6.1
MHD baseline, mean ± SD	17.5 ± 6.6	17.3 ± 6.3	17.8 ± 7.0
BMI, mean ± SD (kg/m^2^)	24.5 ± 4.4	24.4 ± 4.4	24.7 ± 4.5
History of smoking, n (%)	7 (3.6)	4 (4.0)	3 (3.3)
Systolic BP, mean ± SD (mmHg)*	120 ± 9.8	119 ± 10.0	121 ± 9.5
Diastolic BP, mean ± SD (mmHg)*	78 ± 7.2	76 ± 7.8	80 ± 5.9

BP, blood pressure; MMD, monthly migraine days; MHD, monthly headache days. A month is defined as 28 days. Baseline = 28 days before starting treatment. *At baseline, 31 blood pressure measurements were missing (17 for erenumab, 14 for fremanezumab).

### Cerebro-and cardiovascular adverse events

**Figure 2:**
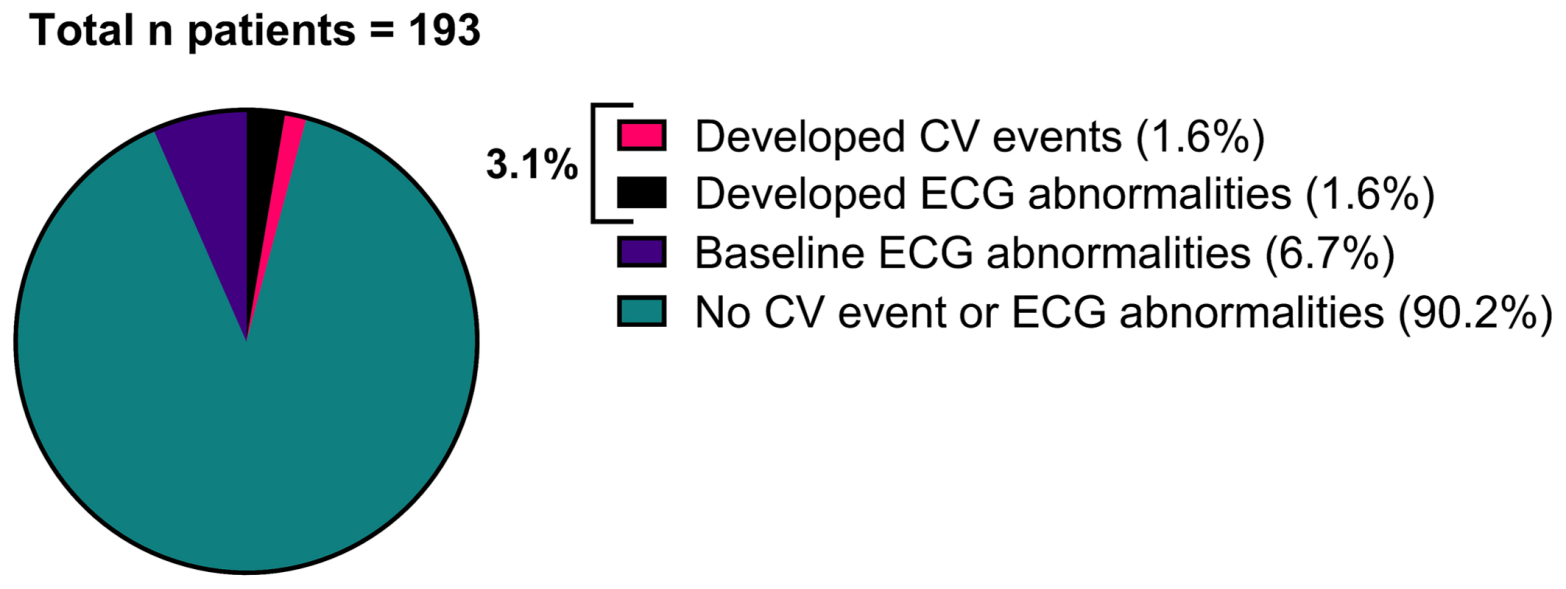
Overview of all patients (*n* = 193), categorized by patients that developed a moderate to severe CV event (*n* = 3), patients that developed ECG abnormalities (*n* = 3), patients that had baseline ECG abnormalities without physical complaints (*n* = 13) and patients that had no CV event or ECG abnormalities (*n* = 174).

Of all patients, 3.11% (6/193) developed an abnormal ECG or CV adverse events during treatment with either erenumab (*n* = 3) or fremanezumab (*n* = 3). At baseline, all these patients had normal SCORE2 risks and BP measurements (Supplementary Table 1). Three of these six patients (1.55%) discontinued their treatment due to a moderate to severe CV events including a cerebellar stroke (*n* = 1), a spontaneous coronary artery dissection (SCAD) (*n* = 1), and pericarditis (*n* = 1) (Table 2). Additionally, the other three (1.55%) patients developed ECG abnormalities without physical complaints during follow-up (Table 3). All six patients had normal ECG readings and did not report any (history of) CV symptoms prior to the start of the study. Furthermore, a distinct subset of 13/193 (6.74%) patients had baseline ECG abnormalities without physical complaints that remained stable throughout the entire study period. None of these patients discontinued treatment due to these abnormalities, as presented in Table 4. An overview of all percentages of CV adverse events is displayed in Figure 2.

**Table 2:** Characteristics of patients with a moderate to severe cardiovascular event during 18 months follow-up.

	Sex	Age (years)	BMI (kg/m^2^)	Diagnosis	CGRP-mAb	SCORE2 (10-year CVD risk)	CVD history	Baseline	3 months	6 months	9 months	12–18 months	Conclusion
1	F	34	20.1	MA	Erenumab	<2.5%	None	Normal	Normal ECG	Normal ECG	SCAD needing PCI	¶	Discontinuation of treatment
2	F	42	29.1	MA	Fremanezumab	<2.5%	None	Normal	Pericarditis	¶	¶	¶	Discontinuation of treatment
3	F	43	22.1	MO	Fremanezumab	<2.5%	None	Normal	Normal ECG	Normal ECG	Normal ECG	Normal ECG, cerebellar stroke (18 m)	Discontinuation of treatment

CVD, cardiovascular disease; CGRP-Mab, Calcitonin Gene Related Peptide – Monoclonal antibody; F, female; MA, migraine with aura; MO, migraine without aura; PCI, percutaneous coronary intervention; SCAD, spontaneous coronary artery dissection. SCORE2 = risk assessment model to estimate the 10-year risk of cardiovascular disease in individuals without previous CVD or diabetes aged 40–69 years in Europe (24), factors included in this prediction model are sex, age, systolic blood pressure, smoking status and non-HDL cholesterol. * = (random) missing data, ¶ = missing due to discontinuation of treatment.

**Table 3:** Characteristics of patients that had a normal ECG at baseline and developed ECG abnormalities without physical complaints during 12 months follow-up.

	Sex	Age (years)	BMI (kg/m^2^)	Diagnosis	CGRP-(R-) Mab	SCORE2 (10 year CVD risk)	CVD history	Baseline	3 months	6 months	9 months	12 months	Conclusion
4	F	40–44	17.3	MO	Erenumab	<2.5%	None	Normal	Normal ECG	Normal ECG	Normal ECG	Negative ST-segments V1-V3	Discontinuation of treatment. Cardiologist: by absence of physical complaints no further analysis
5	F	68	22.5	MO	Fremanezumab	5 to <10%	None	Normal	PVC, mild hypertension	PVC, moderate hypertension	Normal ECG, severe hypertension	RBBB, mild hypertension	Cardiologist: no objection to continuing CGRP-Mab
6	F	49	23.2	MO	Fremanezumab	<2.5%	None	Normal	PVC, mild hypertension	Normal ECG	PVC	Normal ECG	*No intervention*

CVD, cardiovascular disease; CGRP-Mab, Calcitonin Gene Related Peptide – Monoclonal antibody; F, female; LVH, left ventricular hypertrophy; M, male; MA, migraine with aura; MO, migraine without aura; PVC, premature ventricular complex; RBBB, right bundle branch block. SCORE2 = risk assessment model to estimate the 10-year risk of cardiovascular disease in individuals without previous CVD or diabetes aged 40–69 years in Europe (24), factors included in this prediction model are sex, age, systolic blood pressure, smoking status and non-HDL cholesterol. * = (random) missing data, ¶ = missing due to discontinuation of treatment.

**Table 4:** Characteristics of patients with baseline ECG abnormalities without physical complaints during 12 months follow-up.

	Sex	Age (years)	BMI (kg/m^2^)	Diagnosis	CGRP-(R-) Mab	CVD history	Baseline	3 months	6 months	9 months	12 months	Conclusion
1	F	39	20.1	MA	Erenumab	None	Right heart axis	Right heart axis	Right heart axis	Right heart axis	Right heart axis	*No intervention*
2	F	63	27.5	MA	Erenumab	Hypertension	AV-block grade I	AV-block grade I, mild hypertension	AV-block grade I, moderate hypertension	AV-block grade I, mild hypertension	Normal, mild hypertension	*No intervention*
3	M	49	21.6	MA	Erenumab	Congenital aorta valve insufficiency	Left heart axis, LVH	Left heart axis, LVH	Left heart axis, LVH	*	Left heart axis, LVH	*No intervention*
4	M	56	28.7	MO	Erenumab	None	AV-block grade I	AV-block grade I, mild hypertension	AV-block grade I, mild hypertension	AV-block grade I	AV-block grade I, mild hypertension	*No intervention*
5	M	48	21.9	MA	Erenumab	Biphasic P-wave, no abnormalities found on echocardiography	Biphasic P-wave	Biphasic P-wave, moderate hypertension	Biphasic P-wave, mild hypertension	*, severe hypertension	*, severe hypertension	Start of anti-hypertensive medication
6	M	47	22.1	MO	Erenumab	None	RBBB	RBBB, mild hypertension	*	RBBB	RBBB	Cardiologist: no objection to continuing CGRP-R-Mab
7	F	52	23.0	MA	Erenumab	None	Left heart axis	*	Left heart axis	Normal ECG, mild hypertension	Left heart axis	*No intervention*
8	F	61	18.9	MA	Erenumab	None	Right heart axis	Right heart axis	Right heart axis	Right heart axis	Right heart axis	Cardiologist: benign variant
9	F	41	22.5	MO	Fremanezumab	None	Left anterior fascicular block	Left anterior fascicular block	Left anterior fascicular block	Left anterior fascicular block	Left anterior fascicular block	Cardiologist: no objection to continuing CGRP-Mab
10	F	47	24.7	MO	Fremanezumab	Wolff-Parkinson-White (WPW) syndrome	AV-block grade I	Normal ECG	Normal ECG	Normal ECG	Normal ECG	*No intervention*
11	F	26	18.8	MA	Fremanezumab	None	PAC	PAC	PAC	PAC	Left posterior fascicular block	Cardiologist: no objection to continuing CGRP-Mab
12	F	68	19.9	MO	Fremanezumab	None	RBBB	RBBB	RBBB	RBBB	RBBB	Cardiologist: no objection to continuing CGRP-Mab
13	F	49	25.5	MA	Fremanezumab	None	Left heart axis	Left heart axis	Left heart axis	Left heart axis	*	Cardiologist: no objection to continuing CGRP-Mab

CVD, cardiovascular disease; CGRP-Mab, Calcitonin Gene Related Peptide – Monoclonal antibody; F, female; LAH, left atrium hypertrophy; LVH, left ventricular hypertrophy; M, male; MA, migraine with aura; MO, migraine without aura; PAC, premature atrial complex; RBBB, right bundle branch block. * = (random) missing data; ¶ = missing due to discontinuation of treatment.

### Laboratory values

Among the laboratory values no outliers were detected with the principle component analyses.

**Figure 3:**
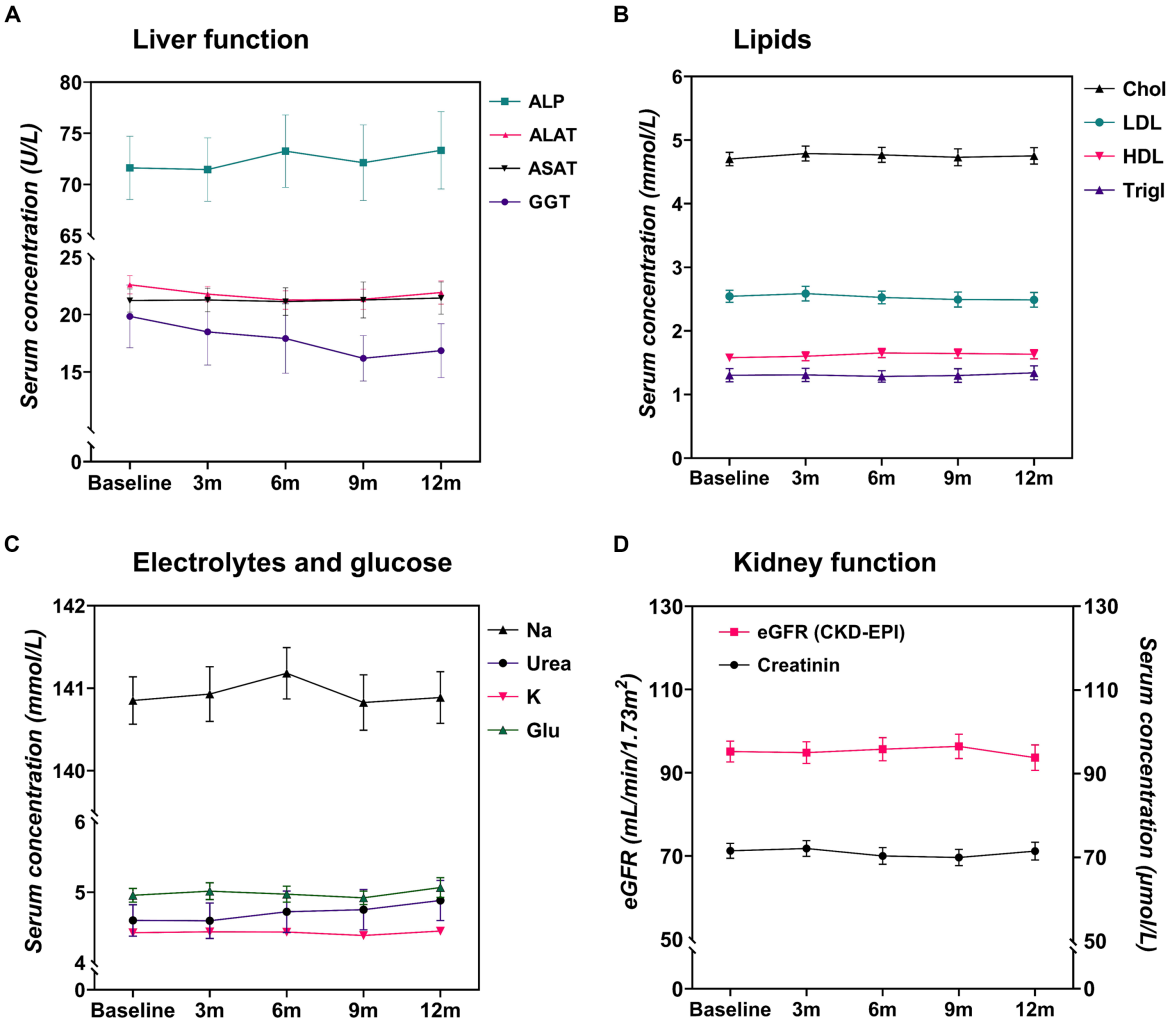
Crude laboratory values (means and 95% confidence intervals) of all patients treated with anti-CGRP monoclonal antibodies (*n* = 193, erenumab and fremanezumab combined). ASAT, aspartate aminotransferase; ALAT, alanine aminotransferase; ALP, alkaline phosphatase; Chol, cholesterol; eGFR, estimated glomerular filtration rate; GGT, gamma-glutamyl transferase; Glu, glucose; HDL, High Density Lipoprotein; K, potassium; LDL, Low Density Lipoprotein; Na, sodium; Trigl, triglycerides.

There was no mean change in ASAT, ALAT and GGT serum levels over time (Figure 3A). Only ALP showed a slight increase at 6 months (*β* = 2.77, 95% CI: 1.07–4.48, *p* = 0.03). Cholesterol, LDL, HDL and triglyceride serum levels remained stable over time (Figure 3B). Similarly, no mean change was found in sodium, potassium, urea and glucose serum levels over time (Figure 3C). Furthermore, there were no mean changes in creatinine serum levels at each time point (Figure 3D). Notably, in the case of eGFR, there was an enhancement in kidney function, characterized by a significant mean increase in eGFR at the 9-month mark (*β* = 3.05, 95% CI: 1.35–4.75, *p* = 0.01). For comprehensive insight, detailed coefficients (95% CI) and *p*-values for each time point are available upon request for all variables.

### ECG conduction times and heart rate

There was no mean change in PR interval time, QRS complex, QTc interval time or heart rate (HR) over time (Supplementary Figure 1). For these conduction times and the heart rate, estimated coefficients (95% CI) and p-values are available upon request.

### Complete case analyses

Out of the initial 193 patients, *n* = 67 were excluded from the analysis due to treatment discontinuation at any time point (Figure 1). We performed a complete case analysis with the remaining 126 patients to handle missing data and investigated the impact on all outcome variables over time. Notably, in the complete case analysis we no longer found an increase in ALP levels at 3 months (*β* = 2.41, 95% CI: 0.36–4.46, *p* = 0.42), nor did we find an elevation of eGFR (CKD-EPI) levels at 9 months (*β* = 2.89, 95% CI: 1.03–4.75, *p* = 0.05).

## Discussion

This study investigated the long-term safety of anti-CGRP(R)-mAbs as migraine treatment. Among all patients, 3.11% developed abnormal ECG or CV adverse events during treatment with erenumab (*n* = 3) or fremanezumab (*n* = 3). Within this group, 1.55% developed moderate to severe CV adverse events that led to treatment discontinuation. These adverse events included cerebellar stroke, SCAD, and pericarditis. The remaining 1.55% developed non-threatening ECG abnormalities without physical complaints. It is noteworthy that these events occurred in patients with no prior hypertension and no prior CV complaints. No clinically meaningful changes were observed in liver, kidney function, lipid, electrolyte, or glucose levels over time.

Migraine itself is associated with an elevated risk of myocardial infarction (OR 2.2, 95% CI 1.7–2.8) and stroke (OR 1.5, 95% CI 1.2–2.1) (25). It is important to note that the absolute risk of CV disease in patients with migraine remains generally low, and is influenced by various factors, including migraine subtype, age, sex and the presence of additional risk factors. In a large population-based study spanning 11.9 years, 697 CV events consisting of both stroke or myocardial infarction were reported among 3,577 women with migraine, resulting in an annual incidence of 1.64% (26). Another population-based study among people with migraine reported cumulative incidences over 19 years for myocardial infarction and ischemic stroke separately. Specifically, for myocardial infarction, there were 25 cases per 1,000 people, which translates to an annual incidence of 0.13%. For ischemic stroke, the study found 45 cases per 1,000 people, resulting in an annual incidence of 0.24% (27).

We recognize the inherent complexity in directly comparing our cohort study with population-based studies. Our selection process may introduce bias by favoring patients with fewer CV risk factors or more severe migraine cases. Additionally, our study encompasses non-ischemic CV events, including pericarditis. Nonetheless, our findings align with these population-based studies. We identified one individual with an ischemic stroke within a 1.5-year follow-up period, resulting in an annual incidence rate of 0.34% for ischemic stroke. To our knowledge, there is no study investigating the association between pericarditis and migraine. Furthermore, although the lifetime prevalence of migraine seems to be elevated in individuals with SCAD compared to the general population (28), there are no studies investigating the incidence of SCAD specifically among patients with migraine. However, a history of migraine is correlated with an increased risk of cervical artery dissection, with an odds ratio of 1.74 (95% CI 1.38–2.19) (29). The precise biological mechanisms underlying this association remain elusive, although genetic analyses have hinted at the existence of common genetic factors influencing vascular structure and function in both migraine and cervical artery dissection (29).

While our findings suggest a comparable annual incidence of 0.34% for ischemic stroke among patients with migraine, it is essential to acknowledge that we cannot definitively exclude the possibility that treatment with anti-CGRP(R)-mAbs might influence the risk of all CV events. Notably, our cohort consisted of healthy individuals with no cardiovascular risk factors at baseline. In contrast, the aforementioned population-based study reporting a one-year incidence of 1.64% included approximately 10% of subjects who were smokers, some had a history of diabetes, and the mean age was approximately 10 years higher compared to our cohort (26). Additionally, the other population-based study included individuals with diabetes (1.37%), hypercholesterolemia (0.73%), hypertension (3.03%), and even other comorbidities such as cancer or liver/renal disease (27). Given these differences, further studies are warranted, particularly those that include control groups matched for CV risk factors and migraine severity. For now, it remains crucial for practitioners to be vigilant, as anti-CGRP(R)-mAbs may potentially increase the severity or impact of such events if they do occur (6). During myocardial infarction (MI) or stroke, there is a release of CGRP, which possesses vasodilatory properties (30, 31). Vasodilation improves the blood flow and oxygen delivery to affected tissue and therefore plays a protective role. Consequently, CGRP may mitigate the extent of tissue damage. Inhibiting CGRP, either through blocking its receptors or capturing the released CGRP before it reaches the receptor, has the potential to exacerbate the deleterious effects of MI or TIA/stroke (5).

The above is especially important since real-world data (RWD) studies revealed elevated BP after exposure to anti-CGRP(R)-mAbs, which was not previously observed in the pivotal randomized clinical trials (20, 21, 32). This discrepancy is most likely due to the dichotomization of the BP outcome variable used in the clinical trials, which focused solely on the occurrence of hypertension. This approach may mask the absolute impact of anti-CGRP(R)-mAbs on BP and potentially downplay the significance of the data (32). Treatment with anti-CGRP(R)-mAbs is associated with an average increase of 5.2 mmHg in systolic BP (21). While some clinicians may perceive this increase as mild, it is crucial to recognize the clinical significance. In fact, a mere 5 mmHg increase in systolic BP will raise the risk of non-fatal CV events by approximately 10% (33). Due to the possibility of many years of treatment, this relative risk increase is clinically important even in participants aged 55 years or younger, which is the age group most represented among patients with migraine (33). Hence, these seemingly modest elevations in BP associated with anti-CGRP(R)-mAbs warrant careful consideration and monitoring to ensure optimal CV health outcomes. Interestingly, patients that developed CV events did not have hypertension, indicating that this factor likely did not contribute to the incidence of CV adverse events. However, as previously emphasized in our earlier publication (21), monitoring of BP remains extremely important. According to the American College of Cardiology and the American Heart Association Task Force, recommended long-term BP targets are ≤130/80 mmHg in patients younger than 65 years and ≤140/90 mmHg in patients 65 years or older (34, 35). Following this stricter blood pressure target of ≤130/80 mmHg, there was one individual that developed pericarditis that had a baseline BP of 126/84 mmHg and would have been excluded from treatment under this guideline. However, all other cases of cardiovascular adverse events and the observed developments of ECG abnormalities in this study had baseline blood pressure readings below the ≤130/80 mmHg threshold. Additionally, it is worth noting that in total, 68 out of 193 patients (35%) in this study had a baseline blood pressure that was higher than 130/80 mmHg but still within the range of ≤140/90 mmHg. According to our current blood pressure guidelines, these patients would not have been eligible for anti-CGRP(R)-mAb treatment unless their blood pressure was actively managed.

A limitation of this study may be some missing data toward the end of the follow-up period, primarily caused by patients discontinuing the treatment. It is important to acknowledge that the occurrence of missing data was not random, as discontinuation may have resulted from adverse events and was therefore possibly associated with an outcome. Therefore, a complete case analysis was added, and the results demonstrated no changes in ASAT, ALAT, GGT, lipid panel, electrolytes, and glucose levels over time and the previously observed changes in ALP and creatinine serum levels were no longer present. In addition, we chose not to include a control group in our study, a decision justified by a single key reason. The inclusion of a control group would not have replicated the findings of pre-existing large population-based studies that already established annual incidence rates of CV events in patients with migraine (26, 27).

A major strength of this study is the utilization of RWD. RWD mirrors the precision, consistency, and verifiability inherent in prospective clinical trials. Unlike experimental studies, RWD-driven observational research dispenses with control groups or manipulated interventions. It relies solely on observed data, capturing patient health statuses and healthcare specifics from diverse sources. The associated Real-World Evidence (RWE) holds great importance, offering a contextual perspective for randomized trials, filling in their data gaps, and addressing broader questions regarding intervention impact in real-life settings. Even in the absence of control groups, RWD and RWE cast light upon healthcare practices, patient outcomes, and the real-world implications of medical interventions. Another strength is that this RWD study excels in incorporating a baseline period and establishing an initial health and CV risk profile. By contrasting treatment period safety outcomes against baseline data, we aimed to identify any notable deviations or adverse effects. The longitudinal design, with over 12 months of follow-up, extends the assessment over time. Nevertheless, we advocate for lengthier RWD studies with similar data collection on CV adverse events for a more comprehensive understanding. For now, we advise to establish the CV risk for each patient prior to the start of treatment with anti-CGRP(R)-mAbs by blood tests, BP measurement and an ECG. After the start of treatment, regular blood tests or ECG measurements may not be indicated unless the patient develops symptomatic complaints. Regular BP monitoring during treatment with CGRP monoclonal antibodies is advised (21).

In conclusion, we identified CV events in 1.55% of patients receiving anti-CGRP(R)-mAbs with 1.5-year follow-up, despite having no CV risk factors or hypertension at baseline. We advise awareness regarding CV events in patients with migraine undergoing CGRP-targeted treatment, not as a confirmation of increased risk but as a proactive measure to address potential multifactorial influences.

### Clinical implications

- 1.6% of patients receiving anti-CGRP(R)-mAbs developed moderate/severe CV events during a 1.5-year follow-up.
- We advise to establish the CV risk for each patient prior to the start of treatment by blood tests, BP measurement and an ECG.
- After the start of treatment, regular blood tests or ECG measurements may not be indicated unless the patient develops symptomatic complaints.
- Regular BP monitoring during treatment with CGRP monoclonal antibodies is advised.

## Data availability statement

The raw data supporting the conclusions of this article will be made available by the authors, without undue reservation.

## Ethics statement

The studies involving humans were approved by nWMO-committee division 3 Leiden University Medical Center. The studies were conducted in accordance with the local legislation and institutional requirements. The ethics committee/institutional review board waived the requirement of written informed consent for participation from the participants or the participants’ legal guardians/next of kin because of the retrospective non-WMO design with data of patients who previously gave written informed consent through protocol P17.232 and the Biobank regulation of the LUMC.

## Author contributions

BA: Conceptualization, Data curation, Formal analysis, Methodology, Project administration, Writing – original draft, Writing – review & editing. NV: Conceptualization, Formal analysis, Methodology, Writing – original draft, Writing – review & editing. JR: Data curation, Formal analysis, Writing – original draft, Writing – review & editing. MO: Writing – review & editing. AM: Conceptualization, Investigation, Methodology, Supervision, Writing – review & editing. GT: Conceptualization, Investigation, Methodology, Supervision, Writing – review & editing.
