# Supplementary material for: Safety considerations in the treatment with anti-CGRP(R) monoclonal antibodies in patients with migraine
Source: Front Neurol. 2024 Apr 29;15:1387044. doi: 10.3389/fneur.2024.1387044 (PMC11089895; doi:10.3389/fneur.2024.1387044)
Supplement: Supplementary file 1 [file Data_Sheet_1.docx]

**Supplementary files**

**eTable 1**. Blood pressure values of patients who developed CV adverse events (Table 2) or ECG abnormalities without physical complaints (Table 3) during follow-up (mmHg).

| **ID** | **Baseline** | **3 months** | **6 months** | **9 months** | **12 months** | **SCORE2 (10 year CVD risk)** | **(Change in) antihypertensive medication** | **CV adverse events or ECG abnormalities** |
| --- | --- | --- | --- | --- | --- | --- | --- | --- |
| 1 | 102/70 | 115/78 | 118/75 | ¶ | ¶ | <2.5% | No | SCAD needing PCI (9 months) |
| 2 | 126/84 | ¶ | ¶ | ¶ | ¶ | <2.5% | No | Pericarditis (3 months) |
| 3 | 103/67 | 110/70 | 122/76 | 112/73 | 96/75 | <2.5% | No | Cerebellar stroke (18 months) |
| 4 | 117/78 | 116/66 | 109/64 | * | 114/75 | <2.5% | No | Negative ST-segments V1-V3 (12 months) |
| 5 | 110/70 | **146**/**98** | **177**/74 | **180**/**100** | **140**/84 | 5 to <10% | No | PVC (3-6 months), hypertension (3-9 months), RBBB (12 months) |
| 6 | 133/84 | **144**/80 | 110/77 | 120/78 | 122/80 | <2.5% | No | PVC (3+9 months) |
| CVD = cardiovascular disease, PCI = percutaneous coronary intervention, SCAD = spontaneous coronary artery dissection, ST-segment = interval between ventricular depolarization and repolarization. SCORE2 = risk assessment model to estimate the 10-year risk of cardiovascular disease in individuals without previous CVD or diabetes aged 40-69 years in Europe^24^, factors included in this prediction model are sex, age, systolic blood pressure, smoking status and non-HDL cholesterol. * = (random) missing data, ¶ = missing due to discontinuation of treatment. | | | | | | | | |

**eFigure 1.** Crude QRS, PR, QTc and HR (means and 95% confidence intervals) of all patients treated with anti-CGRP monoclonal antibodies (n=196, erenumab and fremanezumab combined). QRS = depolarization of the ventricles, PR = time between the P wave (atrial depolarization) and the QRS complex, QTc = corrected QT interval, reflecting the duration of ventricular depolarization and repolarization, HR = heart rate.
